# Everolimus Stabilizes Podocyte Microtubules via Enhancing TUBB2B and DCDC2 Expression

**DOI:** 10.1371/journal.pone.0137043

**Published:** 2015-09-02

**Authors:** Stefanie Jeruschke, Kay Jeruschke, Andrew DiStasio, Sinem Karaterzi, Anja K. Büscher, Perihan Nalbant, Ludger Klein-Hitpass, Peter F. Hoyer, Jürgen Weiss, Rolf W. Stottmann, Stefanie Weber

**Affiliations:** 1 Pediatric Nephrology, Pediatrics II, University Hospital Essen, Essen, Germany; 2 Institute of Clinical Biochemistry and Pathobiochemistry, German Diabetes Center, Duesseldorf, Germany; 3 Divisions of Human Genetics and Developmental Biology, Cincinnati Children's Hospital Medical Center, Cincinnati, Ohio, United States of America; 4 Center for Medical Biotechnology, Molecular Cell Biology, University of Duisburg-Essen, Essen, Germany; 5 Institute of Cell Biology, University of Duisburg‑Essen, Essen, Germany; University of Houston, UNITED STATES

## Abstract

**Background:**

Glomerular podocytes are highly differentiated cells that are key components of the kidney filtration units. The podocyte cytoskeleton builds the basis for the dynamic podocyte cytoarchitecture and plays a central role for proper podocyte function. Recent studies implicate that immunosuppressive agents including the mTOR-inhibitor everolimus have a protective role directly on the stability of the podocyte actin cytoskeleton. In contrast, a potential stabilization of microtubules by everolimus has not been studied so far.

**Methods:**

To elucidate mechanisms underlying mTOR-inhibitor mediated cytoskeletal rearrangements, we carried out microarray gene expression studies to identify target genes and corresponding pathways in response to everolimus. We analyzed the effect of everolimus in a puromycin aminonucleoside experimental *in vitro* model of podocyte injury.

**Results:**

Upon treatment with puromycin aminonucleoside, microarray analysis revealed gene clusters involved in cytoskeletal reorganization, cell adhesion, migration and extracellular matrix composition to be affected. Everolimus was capable of protecting podocytes from injury, both on transcriptional and protein level. Rescued genes included *tubulin beta 2B class IIb* (*TUBB2B)* and *doublecortin domain containing 2* (*DCDC2)*, both involved in microtubule structure formation in neuronal cells but not identified in podocytes so far. Validating gene expression data, Western-blot analysis in cultured podocytes demonstrated an increase of TUBB2B and DCDC2 protein after everolimus treatment, and immunohistochemistry in healthy control kidneys confirmed a podocyte-specific expression. Interestingly, *Tubb2b* ^*brdp/brdp*^ mice revealed a delay in glomerular podocyte development as showed by podocyte-specific markers Wilm’s tumour 1, Podocin, Nephrin and Synaptopodin.

**Conclusions:**

Taken together, our study suggests that off-target, non-immune mediated effects of the mTOR-inhibitor everolimus on the podocyte cytoskeleton might involve regulation of microtubules, revealing a potential novel role of TUBB2B and DCDC2 in glomerular podocyte development.

## Introduction

Podocytes are highly differentiated renal glomerular visceral epithelial cells that cover the outer layer of the glomerular basement membrane playing a crucial role in the regulation of glomerular function [1]. These specialized cells show a complex cellular organization consisting of a cell body, thick primary foot processes, and thin secondary foot processes, which are linked by the glomerular slit diaphragms (SDs) [2]. The sophisticated cell shape of podocytes is maintained by the coordinated intracellular filamentous network of cytoskeletal elements, including microtubules (MTs), intermediate filaments (IFs) and actin filaments (AFs). Physiological podocyte function mainly depends on the dynamic regulation of complex cellular structures, in particular the foot processes. MTs and the actin cytoskeleton seem to coordinately control formation of podocyte foot processes [3]. In particular, MT-rich primary foot processes extending from the cell body split into secondary foot processes containing a dynamic actin meshwork that interacts with the secondary foot processes of neighboring podocytes via Nephrin-linked SDs [3]. Over the past few years, the importance of cytoskeletal components for proper podocyte morphology and glomerular function has emerged from a body of functional data. Genetic studies in glomerular disorders identified several mutated genes encoding proteins associated with the podocyte cytoskeleton such as Nephrin, Podocin, CD2AP, Synaptopodin, alpha-Actinin-4, Inverted formin 2 and TRPC6 [4]. Nonetheless, molecular mechanisms regulating podocyte foot process formation are still poorly understood. Among various intracellular signals, multiple actin based cytoskeletal responses have been established to be mediated by the Rho family small GTPases [5]. In particular, Rac1 and Cdc42 stimulate dynamic protrusions, whereas RhoA together with its effector ROCK control formation of contractile actin-myosin stress fibers [6]. Interestingly, our recent publication revealed that this pathway also mediates the cytoskeletal stabilizing effects of the mTOR inhibitor everolimus (EV) [7].

In contrast to the actin cytoskeleton, the role of MTs in podocyte architecture is still insufficiently studied. Taking advantages of an immortalized murine cell line, Kobayashi and colleagues have previously shown that morphogenesis of podocytes requires proper assembly of MTs as well as their transport by a MT-based motor protein, and is regulated by the extracellular matrix [8,9]. Emerging data from multiple different cell systems suggest a reciprocal crosstalk between the actin regulatory signal transduction pathways and MT-dynamics [10,11]. First, MT polymerization has been associated with activation of the Rho GTPase Rac1 promoting dynamic cell protrusions called lamellipodia [12]. On the other hand, MT de-polymerization activates the RhoA-ROCK pathway via release of the guanine nucleotide exchange factor GEF-H1 [13,14]. In turn, RhoA mediated stress fiber contractility is critically involved in the dynamics of cell-substrate contacts which themselves have been suggested to capture MTs in distinct cellular regions [15,16]. Despite the fact that morphogenesis of podocytes strongly depends on MTs [8,17,18], it is not well understood, how MTs might be coordinated with the actin cytoskeleton to control podocyte behavior and how MT dynamics is affected during podocyte injury.

In the present study we performed microarray analysis using cultured human podocytes treated with EV in a puromycin aminonucleoside (PAN) experimental model of podocyte injury in order to define genes that are strongly associated with cytoskeletal damage. We revealed strong association of *tubulin beta 2B class IIb (TUBB2B)* and *doublecortin domain containing 2 (DCDC2)* with proper podocyte function, two proteins involved in MT formation in neuronal cells but not identified in podocytes so far. We provide direct evidence that PAN-induced disorganization of the actin cytoskeleton and microtubules is associated with downregulation of TUBB2B and DCDC2 in human cultured podocytes and podocyte dysfunction. EV ameliorated podocyte damage by preventing these deregulations. Recently, Stottmann and coworkers described neurodevelopmental defects in mice with a homozygous missense mutation in *Tubb2b*. These homozygous mutants do not survive past birth. They have a profoundly affected brain with significant cortical thinning, largely due to massively increased cell death and in addition show an abnormal proliferation of the basal progenitors [19]. Kidneys of these mutant mice have not been studied so far. Here, we present first data demonstrating a specific expression of TUBB2b in podocytes and a significant delay in glomerular podocyte development in *Tubb2b* ^brdp/brdp^ mice underscoring a role for TUBB2B in proper podocyte function.

## Materials and Methods

### Cell culture

Conditionally immortalized human podocytes were generated by Prof. Dr. Moin A. Saleem (University of Bristol, South Mead Hospital, Bristol, UK) [20]. Culture conditions were described previously [20].

### Animals and kidney preparation

*Tubb2b* mice were maintained as previously indicated [19]. Kidneys were dissected at embryonic day (E) 18.5, 1 day before birth, because homozygous mutant mice did not survive past birth. Tissues were formalin-fixed and paraffin-embedded for immunocytochemistry or glutaraldehyde-fixed for transmission electron microscopy. This study was carried out in strict accordance with the recommendations in the Guide for the Care and Use of Laboratory Animals of the National Institutes of Health. The protocol was approved by the Institutional Animal Care and Use Committee of the Cincinnati Children’s Hospital Medical Center (protocol numbers 1D05052 and IACUC2013-0068). All euthanasia and embryo harvests were performed after isoflurane sedation to minimize animal suffering and discomfort. Animal euthanasia was via cervical dislocation and thoracotomy.

### Human tissue samples

Formalin-fixed, paraffin-embedded and snap-frozen specimens from normal control kidney sections were included in this study. Kidneys were obtained from medical nephrectomy specimens. Written informed consent from the donors were obtained for the use of these samples in research. Kidney tissues were used following the guidelines of the local ethics committee of the Medical Faculty of Essen, Germany (protocol number 09–3954), which specifically approved this study. The tissue samples were not procured from a tissue bank or donation center.

### Experimental design and drug treatment

Podocytes were treated with EV in a PAN-model of podocyte injury as described previously [7].

### RNA isolation from cells

Total RNA was isolated using the RNeasy Mini Kit (Qiagen, Hilden, Germany) according to the manufacturer’s instructions including DNase digestion.

### RNA and protein isolation from snap-frozen tissues

Total RNA and protein were isolated using the AllPrep DNA / RNA / Protein Mini Kit (Qiagen, Hilden, Germany) according to the manufacturer’s recommendations using the TissueLyser (Qiagen, Hilden, Germany) for homogenization and including DNase digestion for RNA-isolation.

### Real-time RT-PCR analysis

1–2 μg of total RNA was reverse-transcribed with random hexamers and SuperScript III First-Strand Synthesis System for RT-PCR (Invitrogen, Karlsruhe, Germany) according to the manufacturer’s instructions.

Quantitative real-time RT-PCR (qPCR) was performed in triplets in 20 μl with 20 ng cDNA, 10 μl SYBR Green JumpStart Taq ready Mix (Sigma-Aldrich, Munich, Germany) and 2.5 pmol of sequence specific primers obtained from biomers.net GmbH, Ulm, Germany. qPCR was performed with StepOnePlus engine (Applied Biosystems, Darmstadt, Germany) and the following conditions: 95°C for 2 min; 40 cycles: 95°C for 30 s; 60°C for 1 min; 72°C for 1 min; followed by melting curve analysis.

qPCR for the endogenous control *18S rRNA* was performed according to the manufacturer’s instructions using the TaqMan Gene Expression Assay Hs99999901_s1 (Applied Biosystems, Darmstadt, Germany) in combination with the TaqMan Fast Universal PCR Master Mix (Applied Biosystems, Darmstadt, Germany).

Relative gene expression was calculated by the 2^−ΔΔCT^ method. Primer sequences are listed in Table 1.

**Table 1:** Primer used for qPCR.

Gene Symbol	Gene Title	Sequence forward 5'-3'	Sequence reverse 5'-3'
COL4A3	Collagen, type IV, alpha 3	GGATTGCCAGGATTTTCTGGT	TGGTACACCGACAAGTCCGTA
DCDC2	Doublecortin domain containing 2	CCAGTCGATCAGAGGCCAG	CTTCAAGGTCACCATTCATTCCT
FN1	Fibronectin 1	CGGTGGCTGTCAGTCAAAG	AAACCTCGGCTTCCTCCATAA
GAPDH	Glyceraldehyde-3-phosphate dehydrogenase	ACAACTTTGGTATCGTGGAAGG	GCCATCACGCCACAGTTTC
LPXN	Leupaxin	CCAACGACTACCACCAACTTT	GCCAGGTCTGGTTCATTGCT
MMP24	Matrix metallopeptidase 24	GCCGGGCAGAACTGGTTAAA	CCCGTAAAACTGCTGCATAGT
MYLIP	Myosin regulatory light chain interacting protein	GCAGGCGACTGGGAATCATAG	CGGTTTCTCAGGTTTAGCCAT
MYO5C	Myosin VC	TCGTGGGCGAGAATGACCT	GGCAACTGCTTGTAAGGATTCA
NRG1	Neuregulin 1	AGAGCCTGTTAAGAAACTCGC	GTCCACTTCCAATCTGTTAGCA
PCDH10	Protocadherin 10	TGGATGGTGGAAGGAGTCTTT	TTCAGCGATATTCCCCACGAA
PCDH17	Protocadherin 17	TGATTGACTCCAACGACAACAG	TGAAAGAGTAGAGCACTTCACCA
PCDH9	Protocadherin 9	AACAGATCCTGACACAGGCTT	TCCAGTCCAAAAACACTCTGC
SYNPO2	Synaptopodin 2	CTCGCCCCTGTCAAGACTG	CCAGGCTGTACCGCTTCTA
TGFB1I1	Transforming growth factor beta 1 induced transcript 1	TACAGCACGGTATGCAAGCC	GCAACCGATCTAGCTCACAGAG
THBS1	Thrombospondin 1	TGCTATCACAACGGAGTTCAGT	GCAGGACACCTTTTTGCAGATG
TUBA1A	Tubulin, alpha 1a	TCGATATTGAGCGTCCAACCT	CAAAGGCACGTTTGGCATACA
TUBB	Tubulin, beta class I	ACCAACCTACGGGGATCTGAA	TTGACTGCCAACTTGCGGA
TUBB2B	tubulin, beta 2B class 2b	GGCACGATGGATTCGGTTAGG	ACACGAAATTGTCTGGTCTGAAG

### Affymetrix expression analysis

Target preparation was performed with 200ng RNA essentially as described in the GeneChip 3’ IVT Express Kit user manual (Affymetrix, Santa Clara, USA). Hybridization, washing and staining of the arrays were done according to the GeneChip Hybridization, Wash, and Stain Kit on the GeneChip Scanner 3000 with G7 update.

Following fragmentation, cRNA was hybridized on Affymetrix GeneChips Human Genome U133_Plus_2.0. GeneChips were washed and stained in the Affymetrix Fluidics Station 450 using the Affymetrix Hybridization, Wash and Stain Kit. Hybridization signals were detected with Affymetrix Gene Chip Scanner 3000 and global scaling was performed by Affymetrix GeneChipOperatingSoftware 1.4 using the MAS5 algorithm. Microarray data of all groups have been published in Gene Expression Omnibus (GEO, http://www.ncbi.nlm.nih.gov/geo/) and are accessible through GEO accession number GSE66107.

For data interpretation we only took those gene products into account that provided present hybridization signals in at least one of the two groups compared. Furthermore, only gene products (probe sets) with fold changes (FC) ≤0.5 or ≥2.0 were considered as significantly, differentially expressed.

To identify differentially expressed targets, we performed an ANOVA test study, in which experimental samples were analyzed against the respective baseline (control) samples using the Partek GS ANOVA algorithm including multiple testing correction.

### Western-blot analysis

Cells were harvested using CelLytic MT-buffer (Sigma-Aldrich, Munich, Germany) according to the manufacturer’s instructions. Lysis-buffer was supplemented with Protease Inhibitor Cocktail (Sigma-Aldrich, Munich, Germany), 10 μg / ml aprotinin (Roche, Mannheim, Germany), 10 μg / ml leupeptin (Roche, Mannheim, Germany) and 2 mM phenylmethanesulfonylfluoride (PMSF, Sigma, Munich, Germany). Isolation was performed at 4°C. Total protein content was measured by Bio-Rad protein assay (Bio.Rad, Munich, Germany). Samples (each > 10 μg protein) were supplemented with Laemmli sample buffer (Bio-Rad, Munich, Germany) and boiled for 10 min at 95°C.

Protein extracts from tissues were obtained as described above. 20 μl of each sample in buffer ALO was boiled for 10 min at 95°C.

Proteins were separated using 10% or 12% Mini-PROTEAN TGX Precast Gels (Bio-Rad, Munich, Germany) and transferred on 0.45 μm PVDF Transfer Membranes (Thermo Scientific, Schwerte, Germany) with a MiniProtean Tetra Cell electrophoresis system (Bio-Rad, Munich, Germany) and a Biometra fastblot B34 blotting device (Biometra, Göttingen, Germany). 15 μl Precision Plus Protein All Blue Standard (Bio-Rad, Munich, Germany) were used as marker. Membranes were incubated with primary antibodies against TUBB2B (1:500; AM09375PU-N, Acris, Herford, Germany), THBS1 (1:500; SAB1401385, Sigma, Munich, Germany), DCDC2 (1:500; SAB2500298, Sigma, Munich, Germany), DCDC2 (1:250; HPA031582, Sigma, Munich, Germany), COL4A3 (1:500; HPA042064, Sigma, Munich, Germany), SYNPO2 (1:500; SAB3500586, Sigma, Munich, Germany), PCDH9 (1:500; HPA015581, Sigma, Munich, Germany) or GAPDH (1:10000; G8795, Sigma, Munich, Germany). Secondary antibodies used were horseradish peroxidase-conjugated goat anti-rabbit IgG (Santa Cruz, Heidelberg, Germany; 1:10000 against COL4A3, SYNPO2 and PCDH9) donkey anti-goat IgG (Santa Cruz, Heidelberg, Germany; 1:10000 against DCDC2) and goat anti-mouse IgG (Santa Cruz, Heidelberg, Germany; 1:20000 against GAPDH, 1:10000 against TUBB2B and THBS1). Signal detection was performed with SuperSignal West Femto Chemiluminescent Substrate (Thermo Scientific, Schwerte, Germany) and visualized by the FUSION FX7 chemiluminescence-system (PEQLAB, Erlangen, Germany) und Fusion-software (PEQLAB, Erlangen, Germany). Intensity of signals was determined using ImageJ software (http://rsbweb.nih.gov/ij/index.html). Densitometric data were normalized to GAPDH-loading control.

### Immunofluorescence and cell imaging of podocytes *in vitro*

For immunofluorescence, podocytes were plated on glass coverslips. After treatment, cells were fixed with 100% icecold methanol (Sigma, Munich, Germany) for 10 min at 4°C, washed with PBS and permeabilized with PBS / 0.5% Triton X-100 / 3% BSA / 3% chicken serum / 3% goat serum for 45 min at room temperature. After washing with blocking buffer (PBS / 0.5% BSA), cells were incubated with the appropriate antibody dilutions. Primary antibodies used were: alpha-Tubulin, rabbit polyclonal antibody ab18251 (1:100; Abcam, Cambridge, UK); TUBB2B, mouse polyclonal antibody AM09375PU-N (1:100; Acris, Herford, Germany) and DCDC2, goat polyclonal antibody SAB2500298 (1:100; Sigma, Munich, Germany).

For staining, cells were incubated with the primary antibody at 4°C overnight followed by the corresponding Alexa Fluor 488 / 594 chicken anti-rabbit IgG (H+L) (alpha-Tubulin), Alexa Fluor 488 / 594 goat anti-mouse IgG (H+L) (TUBB2B) or Alexa Fluor 488 / 594 chicken anti-goat IgG (H+L) (DCDC2) (all 1:1000, Invitrogen, Karlsruhe, Germany) in blocking buffer for 1 h at room temperature. In parallel, nuclei were stained with 4.6-diamidino-2-phenylindole dihydrochloride (DAPI, Sigma, Munich, Germany). For actin staining cells were incubated with phalloidin-TRITC (1:1000, Sigma, Munich, Germany).

Negative controls were performed without primary antibodies.

Fluorescence imaging was performed on a fully automated Nikon ECLIPSE T_i_ microscope controlled by NIS Elements AR 3.2 software (Nikon, Düsseldorf, Germany) equipped with a CoolSNAP HQ2 camera (Photometrics, Tucson, Arizona). Images were acquired using a 20x phase contrast objective with appropriate filter sets. Image processing and analysis was performed with ImageJ (http://rsbweb.nih.gov/ij/index.html) and Adobe Photoshop software (Adobe Systems, Mountain View, CA).

### Immunohistochemistry staining of tissues

Immunohistochemistry on formalin-fixed, paraffin-embedded tissues was performed using the Labeled StreptAvidin Biotin kit from DAKO according to the manufacturer’s recommendations. Briefly, paraffin-embedded tissues were sectioned at 5 μm. Sections were deparaffinized in xylene and rehydrated in graded ethanols. After blocking endogenous peroxidase by incubation in 3% hydrogen peroxide (DAKO, Hamburg, Germany) antigen demasking procedure was done with Heat Induced Epitope Retrieval (HIER) in Target retrieval solution pH 6 (DAKO, Hamburg, Germany), using a pressure boiler as heat source for 10 min (TUBB2B, DCDC2, NPHS2, NPHS1 and SYNPO) or with 1 mg / ml protease at 38°C for 10 min (WT1). After blocking of unspecific sites (0,1% Avidin, 0,01% Biotin and Protein Block Serum-free; all obtained from DAKO, Hamburg, Germany), sections were stained with the first antibody for 2 h diluted in Antibody Diluent with Background Reducing Components (DAKO, Hamburg, Germany) followed by incubation with the biotinylated secondary antibody (30 min; DAKO, Hamburg, Germany) and the streptavidin-peroxidase reagent (30 min, DAKO, Hamburg, Germany) and finally 3,3'-diaminobenzidine (DAB; DAKO, Hamburg, Germany) used as the chromogen. Sections were counterstained with Mayer’s Hematoxylin (DAKO, Hamburg, Germany), mounted with Immu-Mount (Thermo Scientific, Schwerte, Germany), and coverslipped. Negative controls consisted of consecutive tissue sections of each case in which the primary antibody was omitted.

Primary antibodies were as follows: TUBB2B, mouse polyclonal antibody AM09375PU-N (1:100; Acris, Herford, Germany); DCDC2, rabbit polyclonal antibody HPA031582 (1:200; Sigma, Munich, Germany); WT1, mouse monoclonal antibody Clone 6F-H2 (1:50; DAKO, Hamburg, Germany); NPHS2, rabbit polyclonal antibody ab93650 (1:200; Abcam, Cambringe, UK); NPHS1, rabbit polyclonal antibody SAB3500770 (1:200; Sigma, Munich, Germany) and SYNPO, goat polyclonal antibody sc-21537 (1:100; Santa Cruz, Heidelberg, Germany).

In addition, periodic acid-Schiff (PAS) staining (Sigma, Munich, Germany) was performed according to the manufacturer’s instructions.

Imaging was performed on a Leica DM RBE microscope (Leica Microsystems, Wetzlar, Germany) equipped with an Olympus DP73 camera (Olympus, Hamburg, Germany) using cellSens Dimension 1.7 software (Olympus Soft Imaging Solutions, Münster, Germany). Images were acquired with a 10x, 20x or 40x PL APO objective. Image processing and analysis was performed using ImageJ (http://rsbweb.nih.gov/ij/index.html) and Adobe Photoshop software (Adobe Systems, Mountain View, CA).

### Tissue Sampling and Processing for Electron Microscopy

Upon initial dissection at embryonic day (E) 18.5, 1 day before birth, mice kidneys were fixed at room temperature in 2.5% glutaraldehyde in PBS. Repositioning was performed in 2.5% glutaraldehyde in 0.19 M cacodylate buffer at pH 7.4. Samples were postfixed in 1% Osmium tetroxide in 0.19 M cacodylate buffer, pH 7.4, for 2 hours. Tissues were stained en bloc in 2% uranyl acetate in 0.05 M maleate buffer, pH 4.6. Specimens then were dehydrated in graded ethanols and embedded in epoxy resin [21]. Toluidine-blue stained semithin sections were used to search for glomeruli. Ultrathin sections were picked up onto Formvar carbon–coated grids, stained with lead citrate, and analyzed with a transmission electron microscope (TEM 910; Zeiss, Oberkochen, Germany) equipped with a cooled CCD digital camera (TRS, Moorenweis, Germany). During TEM studies, 4–11 glomeruli were captured randomly in each of two different wild types and *Tubb2b* ^*brdp/brdp*^ kidneys.

### Statistical analysis

Values from multiple experiments were expressed as means ± SD. Statistical analysis was assessed using Student’s T-Test. Statistical significance was defined as p < 0.05.

## Results

### EV treatment modulates the transcriptome of injured human podocytes

To identify molecules involved in mechanisms underlying EV-mediated protective activity during podocyte injury, we first generated microarray expression data from MeOH (solvent for EV), EV, PAN+MeOH and PAN+EV treated human differentiated podocytes. The Affymetrix GeneChip Human Genome U133_Plus_2.0 was used for transcriptomic analyses with three biological replicates for each treatment. Overall, PAN treatment (PAN+MeOH) caused differential regulation of a substantial number of genes as compared to solvent control (MeOH). Expression of a large portion of these transcripts (195 differentially expressed genes) was partially rescued in the combination with EV (PAN+EV) with more than two-fold changes between the two given treatments (S1 Table). We identified 73 transcripts decreased after PAN treatment and increased in the combination with EV at the same time as well as 122 transcripts increased in PAN+MeOH and decreased in PAN+EV (fold change > 2.0; p-value < 0.05). Critically, among those transcripts, an accumulation of genes involved in the regulation of microtubules was detected.

Table 2 lists selected results from the microarray analysis of podocyte gene expression in PAN+MeOH treated cells compared to solvent control (MeOH) and PAN+EV samples compared to PAN+MeOH. The top of Table 2 contains genes with a significant decrease in expression following PAN+MeOH treatment when compared with solvent only (MeOH). Interestingly, expression of several genes in this group is substantially elevated upon treatment with PAN in combination with EV (PAN+EV). Several of those genes encode proteins playing important roles in cytoskeletal organization and stability, including *TUBB2B*, *DCDC2*, *MYO5C*, *TUBB* and *TUBA1A*. Others that were elevated with EV encode proteins involved in cell-cell adhesion (*THBS1*, *TGFB1I1*, *NRG1*, *FN1*), migration (*DCDC2*, *MMP24*, *FN1*) and extracellular matrix composition (*COL4A3*). Interestingly, the expression of the three major components of MTs *tubulin beta class I* (*TUBB*), *tubulin beta 2B class IIb* (*TUBB2B*) and *tubulin alpha 1a* (*TUBA1A*) was decreased in PAN+MeOH samples as compared to MeOH alone but substantially increased in the presence of EV. The bottom of Table 2 depicts genes that were up-regulated with PAN+MeOH and significant down-regulated in PAN+EV treated cells. In particular, we found accumulation of genes that encode proteins associated with cytoskeletal organization and stability such as *SYNPO2* and *MYLIP* as well as cell adhesion including *LPXN*, *PCDH9*, *PCDH10* and *PCDH17*. Strikingly, the expression of *PCDH9*, -*10* and -*17* (*protocadherin 9*, *-10*, *-17*) was increased up to tenfold when treated with PAN+MeOH as compared to MeOH alone and strongly decreased in combination with EV, the largest fold-changes measured. These genes are known to encode adhesion proteins that mediate cell-cell adhesion in neural tissues [22,23]. Thus, such strong differential regulation of expression levels suggests a potential critical role in podocyte adhesion as well.

**Table 2:** Affymetrix gene expression data selected according to function.

Probeset	Gene Symbol	Gene Title	PAN+MeOH vs. MeOH		PAN+EV vs. PAN+MeOH		Function
			p < 0.05	FC > 1.5	p < 0.05	FC > 1.5	
** **	** **	**Decreased in PAN+MeOH / Increased in PAN+EV**	** **	** **	** **	** **	
214023_x_at	TUBB2B	tubulin, beta 2B	1.83E-08	-19.00	1.45E-05	4.23	cytoskeletal organization / stability
201109_s_at	THBS1	thrombospondin 1	3.98E-07	-13.51	1.17E-03	2.53	adhesion
222925_at	DCDC2	doublecortin domain containing 2	3.71E-09	-6.59	4.71E-06	2.28	cytoskeletal organization / stability, migration
218966_at	MYO5C	myosin VC	3.05E-07	-5.80	2.65E-04	2.25	cytoskeletal organization / stability
212320_at	TUBB	tubulin, beta	6.07E-09	-5.47	8.81E-06	2.05	cytoskeletal organization / stability
209651_at	TGFB1I1	transforming growth factor beta 1 induced transcript 1	2.95E-08	-5.06	4.58E-05	1.95	adhesion
222073_at	COL4A3	collagen, type IV, alpha 3	3.06E-06	-4.80	1.67E-04	3.35	extracellular matrix composition
206343_s_at	NRG1	neuregulin 1	6.12E-07	-3.18	9.39E-05	2.14	adhesion
209118_s_at	TUBA1A	tubulin, alpha 1a	7.33E-09	-2.86	2.52E-06	1.98	cytoskeletal organization / stability
213171_s_at	MMP24	matrix metallopeptidase 24 (membrane-inserted)	3.36E-06	-2.42	1.41E-03	1.56	migration
214702_at	FN1	fibronectin 1	2.14E-05	-1.78	1.23E-03	1.50	adhesion, migration
		**Increased in PAN+MeOH / Decreased in PAN+EV**					
227662_at	SYNPO2	synaptopodin 2	6.66E-05	2.11	9.69E-05	-2.96	cytoskeletal organization / stability
216250_s_at	LPXN	leupaxin	3.30E-06	2.40	8.17E-05	-2.23	adhesion, migration
223130_s_at	MYLIP	myosin regulatory light chain interacting protein	3.77E-08	4.86	1.18E-05	-2.57	cytoskeletal organization / stability
219737_s_at	PCDH9	protocadherin 9	5.41E-08	9.46	6.24E-06	-5.69	adhesion
228635_at	PCDH10	protocadherin 10	1.06E-06	13.92	2.12E-04	-4.94	adhesion
228863_at	PCDH17	protocadherin 17	2.30E-08	17.66	2.61E-06	-10.52	adhesion

Genes were selected based on their putative roles their products may play in stabilizing podocyte cytoskeleton or adhesion and on the intensity of the EV rescue effect. PAN = puromycin aminonucleoside. EV = everolimus. MeOH = methanol, solvent for EV. p = p-value. FC = fold change.

Quantitative RT-PCR (qPCR) was initially used to verify selected microarray results. We selected all 17 genes of Table 2 on the basis of their possible contribution to the functional endpoint of the study (i.e. increased cytoskeletal stability and cell adhesion with EV). These included 11 upregulated and 6 downregulated genes in the presence of EV. Table 3 lists the qPCR results with PAN+EV treated cells compared to PAN+MeOH. In agreement with the microarray, 16 genes were differentially regulated as measured with qPCR from which 12 reached statistical significance.

**Table 3:** qPCR analysis of human podocyte gene expression: Verification of selected microarray results.

Gene Symbol	Gene Title	PAN+MeOH vs. MeOH			PAN+EV vs. PAN+MeOH			Function
		FC	p-value	FC Array*	FC	p-value	FC Array*	
COL4A3	Collagen, type IV, alpha 3	-1.3	ns	-4.8	2.6	0.002	3.3	extracellular matrix composition
DCDC2	Doublecortin domain containing 2	-3.9	0.001	-6.6	2.4	0.004	2.3	cytoskeletal organization / stability, migration
FN1	Fibronectin 1	-2.0	0.03	-1.8	-1.0	ns	1.5	adhesion, migration
LPXN	Leupaxin	5.6	0.01	2.4	-2.2	0.03	-2.2	adhesion, migration
MMP24	Matrix metallopeptidase 24	-1.6	0.01	-2.4	3.0	ns	1.6	migration
MYLIP	Myosin regulatory light chain interacting protein	15.7	0.01	5.3	-2.3	0.04	-2.6	cytoskeletal organization / stability
MYO5C	Myosin VC	-4.1	0.002	-5.8	2.8	0.009	2.3	cytoskeletal organization / stability
NRG1	Neuregulin 1	-1.6	ns	-3.2	1.2	ns	2.1	adhesion
PCDH10	Protocadherin 10	71.1	0.009	13.9	-4.6	0.01	-4.9	adhesion
PCDH17	Protocadherin 17	371.4	0.005	17.7	-12.0	0.006	-10.5	adhesion
PCDH9	Protocadherin 9	32.7	0.02	9.5	-6.7	0.02	-5.7	adhesion
SYNPO2	Synaptopodin 2	1.9	ns	2.1	-2.4	0.04	-3.0	cytoskeletal organization / stability
TGFB1I1	Transforming growth factor beta 1 induced transcript 1	-1.7	0.05	-5.1	2.9	0.008	2.0	adhesion
THBS1	Thrombospondin 1	-10.8	0.0003	-13.5	2.5	0.03	2.5	adhesion
TUBA1A	Tubulin, alpha 1a	-3.8	0.0001	-2.9	1.6	0.03	2.0	cytoskeletal organization / stability
TUBB	Tubulin, beta class I	-3.9	0.0007	-5.5	2.2	0.004	2.0	cytoskeletal organization / stability
TUBB2B	tubulin, beta 2B class 2b	-13.5	0.00008	-19.0	5.8	0.003	4.2	cytoskeletal organization / stability

PAN = puromycin aminonucleoside. EV = everolimus. MeOH = methanol, solvent for EV. p = p-value. FC = fold change.

* = p < 0.05.

### EV protects components of cytoskeletal stability and podocyte adhesion

**Fig 1:**
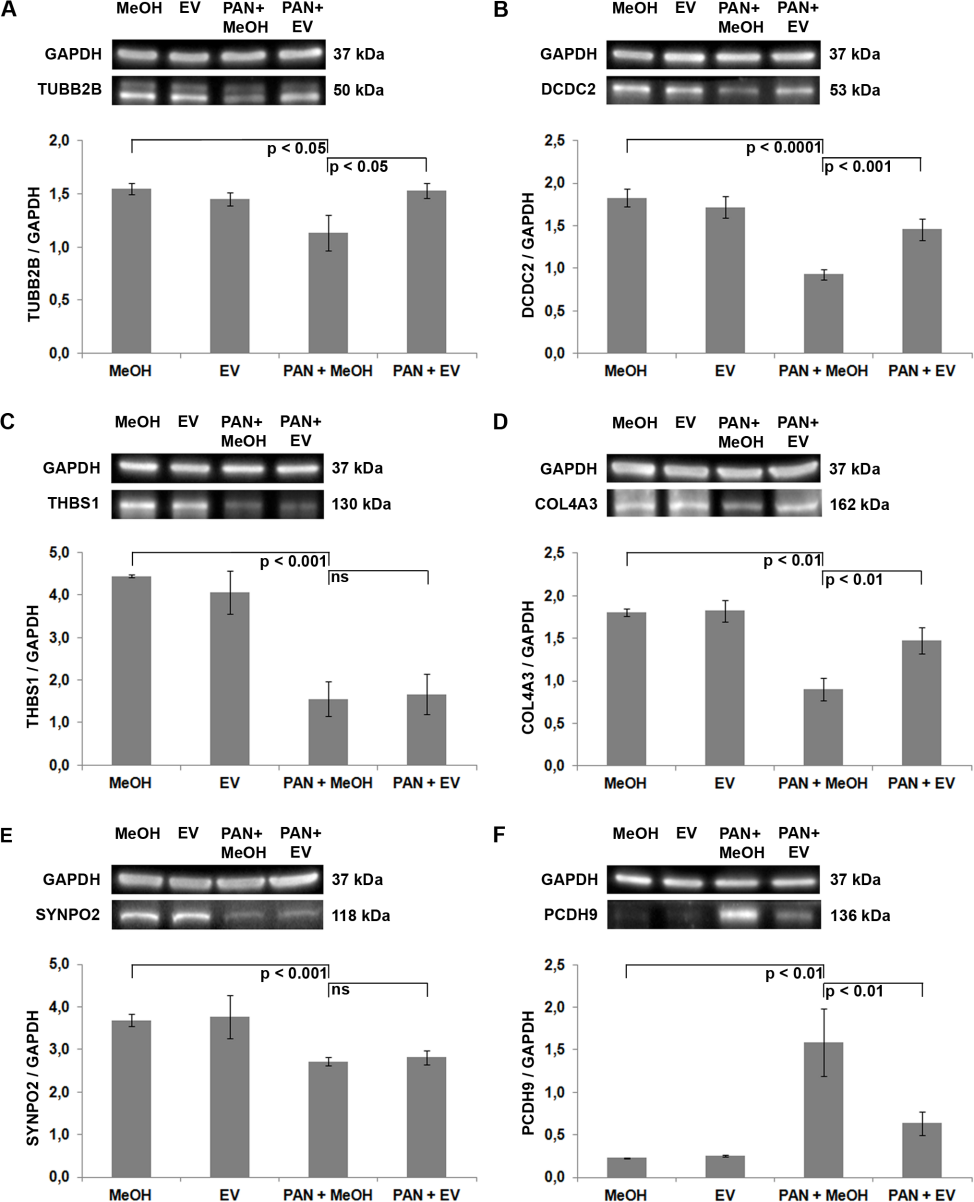
Western-blot analysis of human podocytes: Verification of selected microarray results. Quantification of total TUBB2B (A), DCDC2 (B), THBS1 (C), COL4A3 (D), SYNPO2 (E) and PCDH9 (F) protein. GAPDH = loading control. For quantification, total protein was normalized with respect to GAPDH (representative example from 3 independent experiments). PAN = puromycin aminonucleoside. EV = everolimus. MeOH = methanol, solvent for EV. p = p-value. Data are means ± SD. ns = not significant.

To further confirm the results of the microarray analysis, we performed Western-blot analysis of TUBB2B, DCDC2, THBS1, COL4A3, SYNPO2 and PCDH9 in cultured podocytes selected due to their possible roles in podocytes (Fig 1). We measured the levels of these differentially expressed gene products after PAN treatment and in the presence of EV. PAN dramatically reduced the amount of TUBB2B, DCDC2, THBS1, COL4A3 and SYNPO2 protein (Fig 1A–1E). Addition of EV significantly restored the protein levels of TUBB2B, DCDC2 and COL4A3, without altering THBS1 and SYNPO2 expression. In contrast, PAN lead to an elevated PCDH9 expression, whereas EV tempered the increase of PCDH9 (Fig 1F). Taken together, with the exception of SYNPO2 protein expression, these findings confirm the microarray results.

**Fig 2:**
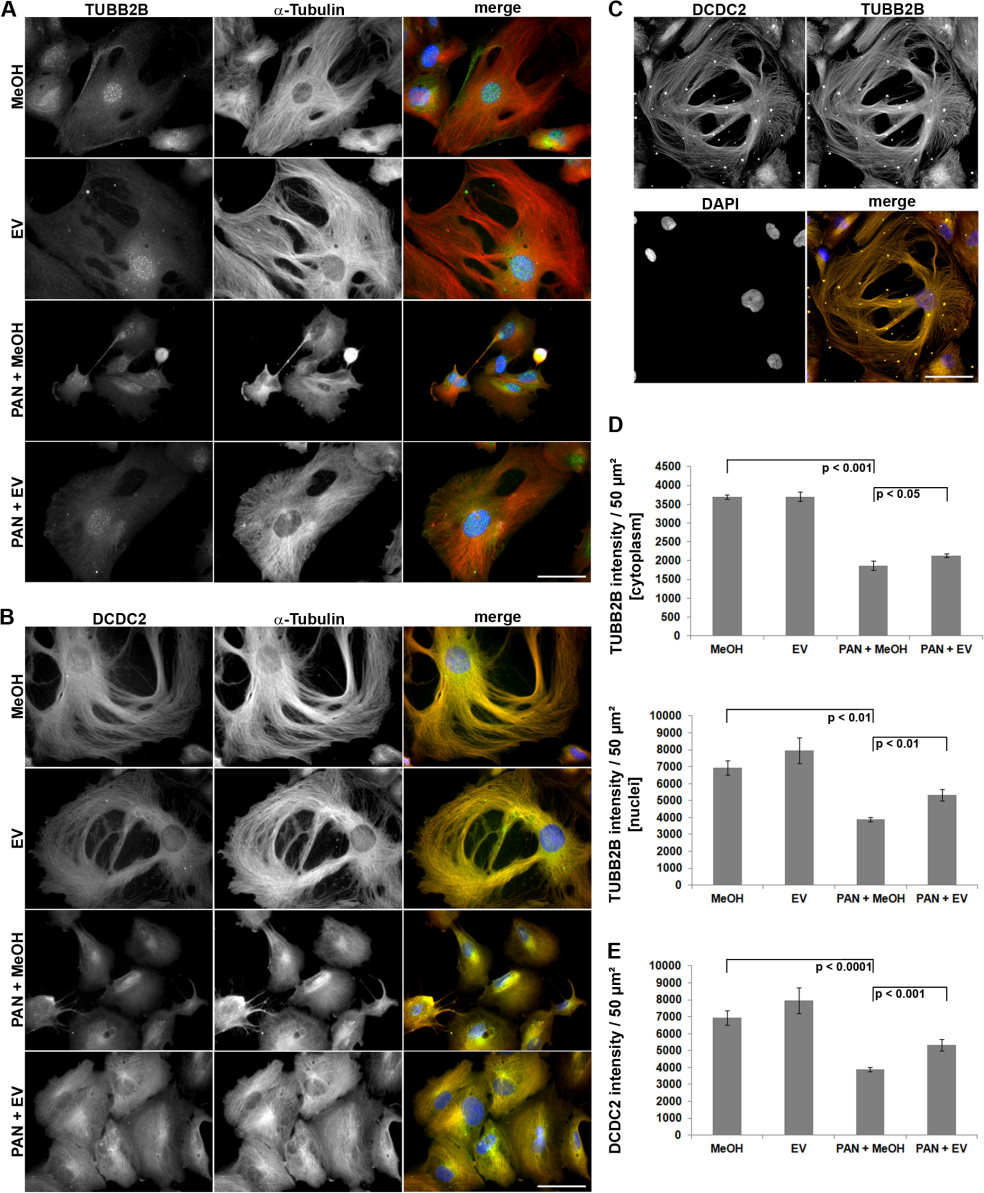
Immunofluorescence analysis of human podocytes: Verification of selected microarray results. (A+B) alpha-tubulin, TUBB2B and DCDC2 images are presented in gray scale for maximum contrast. The merge image depicts TUBB2B and DCDC2 in green and alpha-tubulin in red. DAPI was used to visualize nuclei (blue). PAN-treated cells are smaller compared to control cells, due to alterations of the actin cytoskeleton and cell-substrate adhesion dynamics [7]. (C) Double staining of TUBB2B (red) and DCDC2 (green) of untreated control cells (D+E) Quantification of TUBB2B- and DCDC2 staining intensity with Image J software (n = 3 experiments, ≥ 10 cells per condition). PAN = puromycin aminonucleoside. EV = everolimus. MeOH = methanol, solvent for EV. p = p-value. Data are means ± SD. Scale bar = 50 μm.

The subcellular localization of tubulin beta 2B class IIb (TUBB2B) and doublecortin domain containing 2 (DCDC2), both known to be involved in MT assembly, was subsequently assessed by immunocytochemical stainings in podocytes (Fig 2). We characterized TUBB2B protein localization both in the cytoplasm and in the nucleus of differentiated podocytes (Fig 2A). The nuclear pattern of TUBB2B appeared punctate whereas the cytoplasmic protein colocalized with the alpha-tubulin staining. DCDC2 protein was restricted to the cytoplasm and also colocalized with alpha-tubulin (Fig 2B). Immunostaining of TUBB2B and DCDC2 revealed a colocalization of the two proteins on filamentous structures reminiscent of microtubules (Fig 2C).

In addition to the cellular localization we quantified the staining intensity of TUBB2B and DCDC2 in all treatment groups. This data revealed a reduced expression of TUBB2B in PAN-treated podocytes when compared to MeOH-treated cells, for both the nuclear and the cytoplasmic pattern. Addition of EV significantly restored the staining intensity of TUBB2B in the cytoplasm and to a greater extend in the nuclei (Fig 2D). In line with this data, the expression of DCDC2 was also significantly restored by EV (Fig 2E).

### TUBB2B and DCDC2 are expressed in human kidney tissues

**Fig 3:**
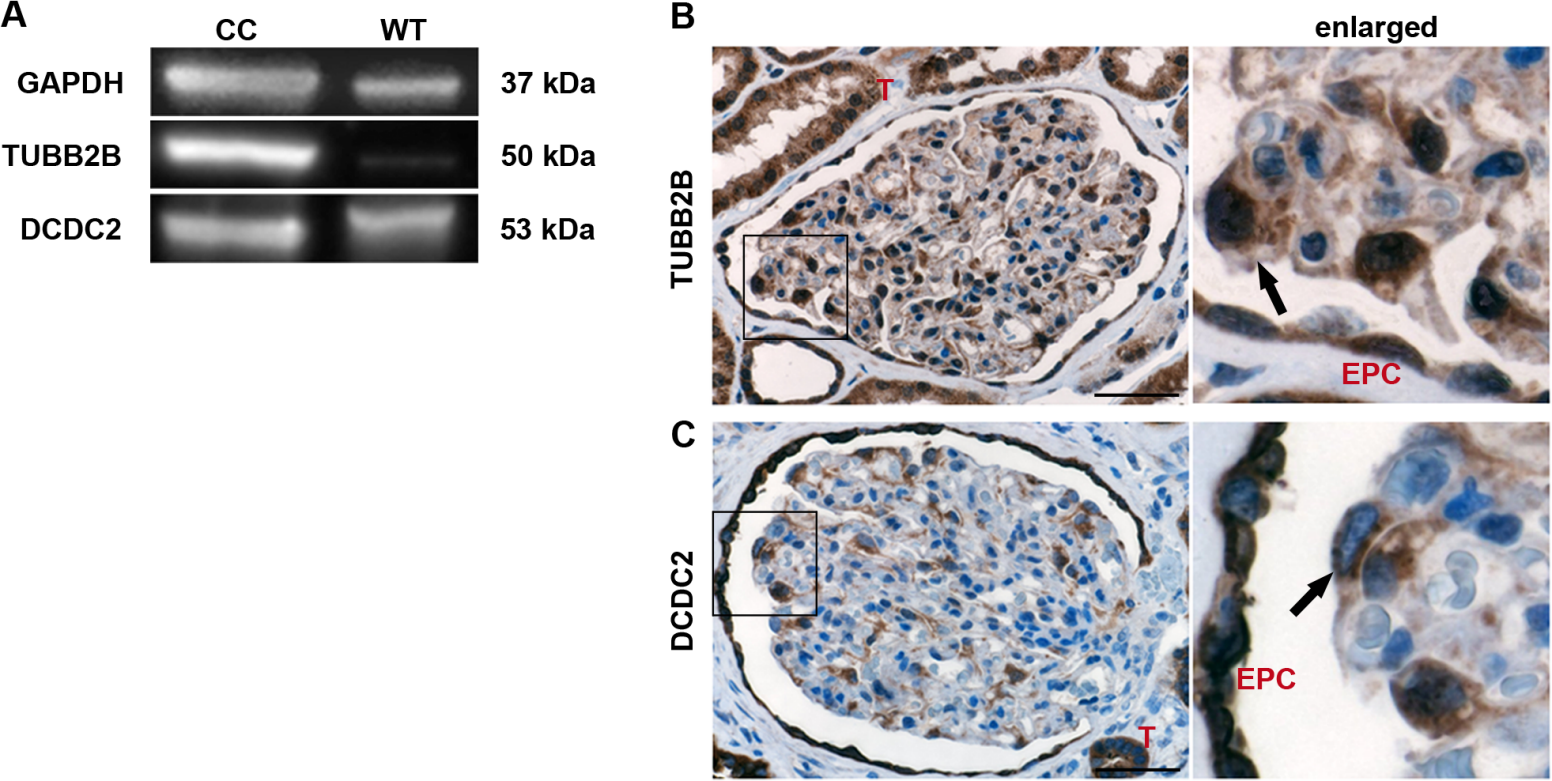
TUBB2B and DCDC2 are expressed in human kidneys. (A) Western-blot analysis to measure the existence of TUBB2B and DCDC2 in human kidneys. GAPDH = loading control. CC = Podocyte cell culture. WT = Wild type kidney. (B+C) Immunohistochemical staining of TUBB2B and DCDC2 in healthy human kidneys. 3,3'-diaminobenzidine (DAB) was used as chromogen (brown staining) and nuclei were stained with hematoxylin (blue). The black arrows mark a nuclear as well as cytoplasmic staining of TUBB2B and a cytoplasmic staining of DCDC2 in podocytes. A negative control was performed without the primary antibody (S1 Fig). T = tubule. EPC = parietal epithelial cell. Scale bar = 50 μm.

To confirm the presence of TUBB2B and DCDC2 *in vivo* in normal human kidney tissues we performed qPCR and Western-blot analyses from extracts received from total kidneys. Both were present on RNA level (data not shown) as well as on protein level (Fig 3A).

To evaluate the localization of both proteins within the kidney *in vivo*, immunohistochemical stainings for TUBB2B and DCDC2 were performed in healthy control human kidneys (Fig 3B+3C). TUBB2B showed distinct cytoplasmic and nuclear staining in tubules, podocytes and epithelial parietal cells (EPCs) (Fig 3B). Pronounced cytoplasmic DCDC2 expression was observed in EPCs, whereas lower levels of DCDC2 were found in podocytes (Fig 3C).

### Delay in glomerular development in mutant *Tubb2b* mice

Recently, Stottmann et al. described a homozygous mouse model expressing a missense mutation in *Tubb2b* [19]. To define the effect of *Tubb2b* ^brdp/brdp^ on glomerular ultrastucture we compared kidneys from E18.5 *Tubb2b* ^brdp/brdp^ and *Tubb2b* ^+/+^ mice by light microscopy and by TEM studies.

**Fig 4:**
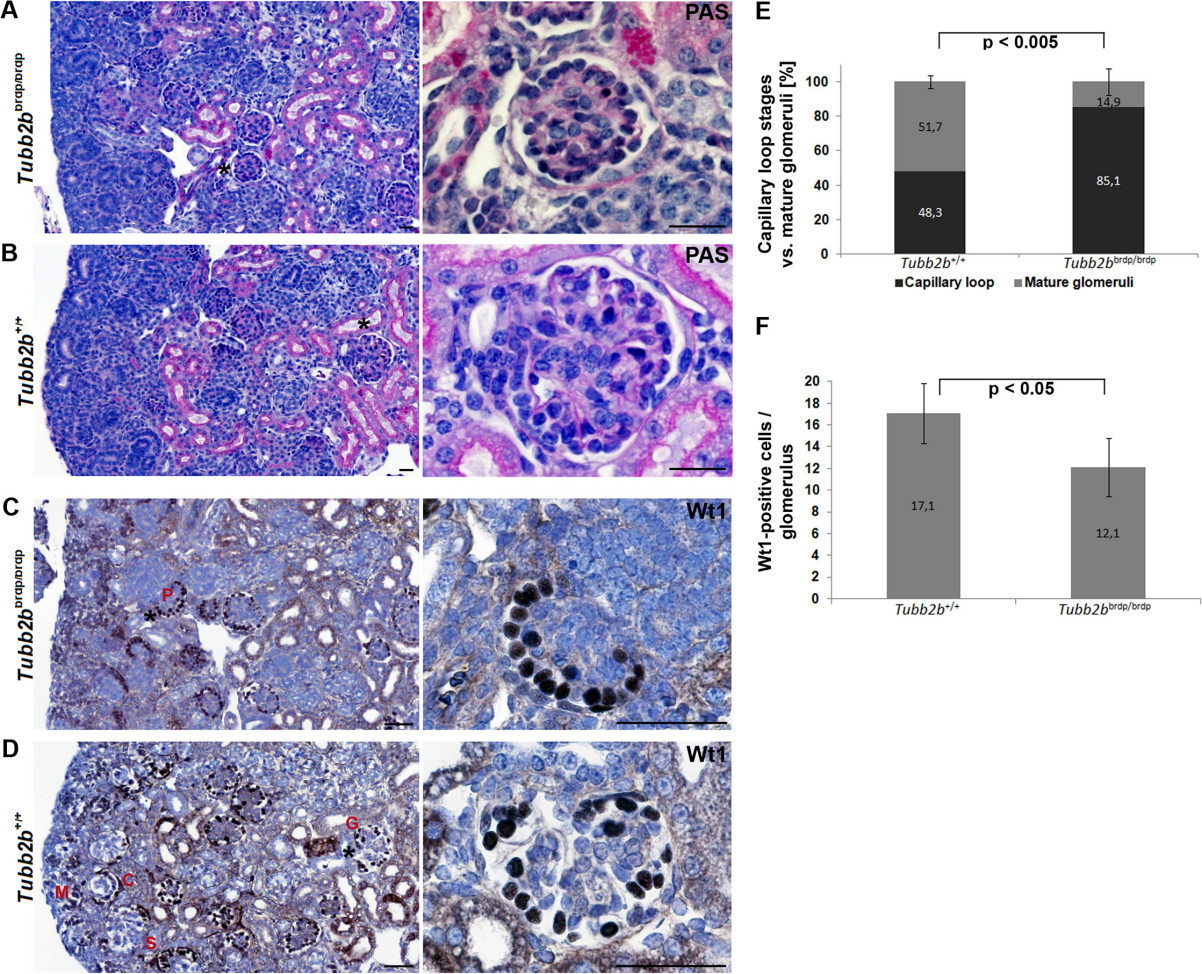
Periodic acid-Schiff and Wilm’s tumour 1 staining of wild type and *Tubb2b* ^brdp/brdp^ mouse kidneys (E18.5). (A+B) Glomeruli were stained with periodic acid-Schiff to highlight basement membranes of glomerular capillary loops and tubular epithelium. (A) *Tubb2b* ^brdp/brdp^ mice often show a lack in glomerular tuft and capillary lumen development. (B) The capillary loops of the wild type glomeruli are well-defined and thin. Scale bars = 25 μm. (C+D) Wilm’s tumour 1 (Wt1) expression was detectable by immunohistochemistry (dark-brown nuclear staining results from 3,3'-diaminobenzidine). Nuclei were stained with hematoxylin (blue). (C) *Tubb2b* ^brdp/brdp^ kidneys show a specific Wt1 staining in podocytes (P) arranged in a string of pearls-like pattern at the periphery of the glomerulus, characteristic of an early developmental stage. (D) In the developing wild type kidney, Wt1 is expressed in mesenchymal cells that are starting the mesenchymal-to-epithelial transition (condensing metanephric mesenchyme (M)), in early epithelial structures (comma- (C) and S-shaped (S) bodies) and in fully differentiated epithelial cells (glomerular podocytes (G)). Black asterisks: Enlarged section areas on the right. Scale bars = 50 μm. (E) Percentage of capillary loop stages versus mature glomeruli in 3 mutant and 4 wild type animals. For each animal, 15 to 30 capillary loop stages / mature glomeruli were counted. Data are means ± SD. (F) Average number of Wt1-positive cells per glomerulus in 3 mutant and 4 wild type animals. 15 to 30 glomeruli / animal were counted. Data are means ± SD.

Light microscopy examination of *Tubb2b* ^brdp/brdp^ glomeruli revealed a significant delay in glomerular tuft and capillary lumen development (Fig 4A), whereas wild type *Tubb2b* ^+/+^ glomeruli had age-dependent normally developed glomeruli with open capillary loops (Fig 4B).

To further explore the cellular alterations underlying the glomerular phenotype observed in *Tubb2b* ^brdp/brdp^ mice, we examined the expression of the podocyte-specific protein Wilm’s tumour 1 (Wt1). The expression pattern of Wt1 in *Tubb2b* ^brdp/brdp^ kidneys revealed a delay in podocyte maturation with podocytes arranged in a string of pearls-like pattern at the periphery of the glomerulus (Fig 4C). In the developing wild type kidney, Wt1 expression is detectable in mesenchymal cells that are starting the mesenchymal-to-epithelial transition (condensing metanephric mesenchyme), in early epithelial structures (comma- and S-shaped bodies) as well as in differentiated epithelial cells (glomerular podocytes) (Fig 4D). To confirm the podocyte maturation defect in *Tubb2b* ^brdp/brdp^ kidneys the percentage of capillary loop stages versus mature glomeruli and the average number of Wt1-positive cells per glomerulus were analyzed. Indeed, a significantly increased number of capillary loop stages in *Tubb2b* ^brdp/brdp^ kidneys compared to mature glomeruli was found (Fig 4E). In addition, significantly less Wt1-positive cells per glomerulus were detected in *Tubb2b* ^brdp/brdp^ kidneys as compared to wild type mice (Fig 4F).

**Fig 5:**
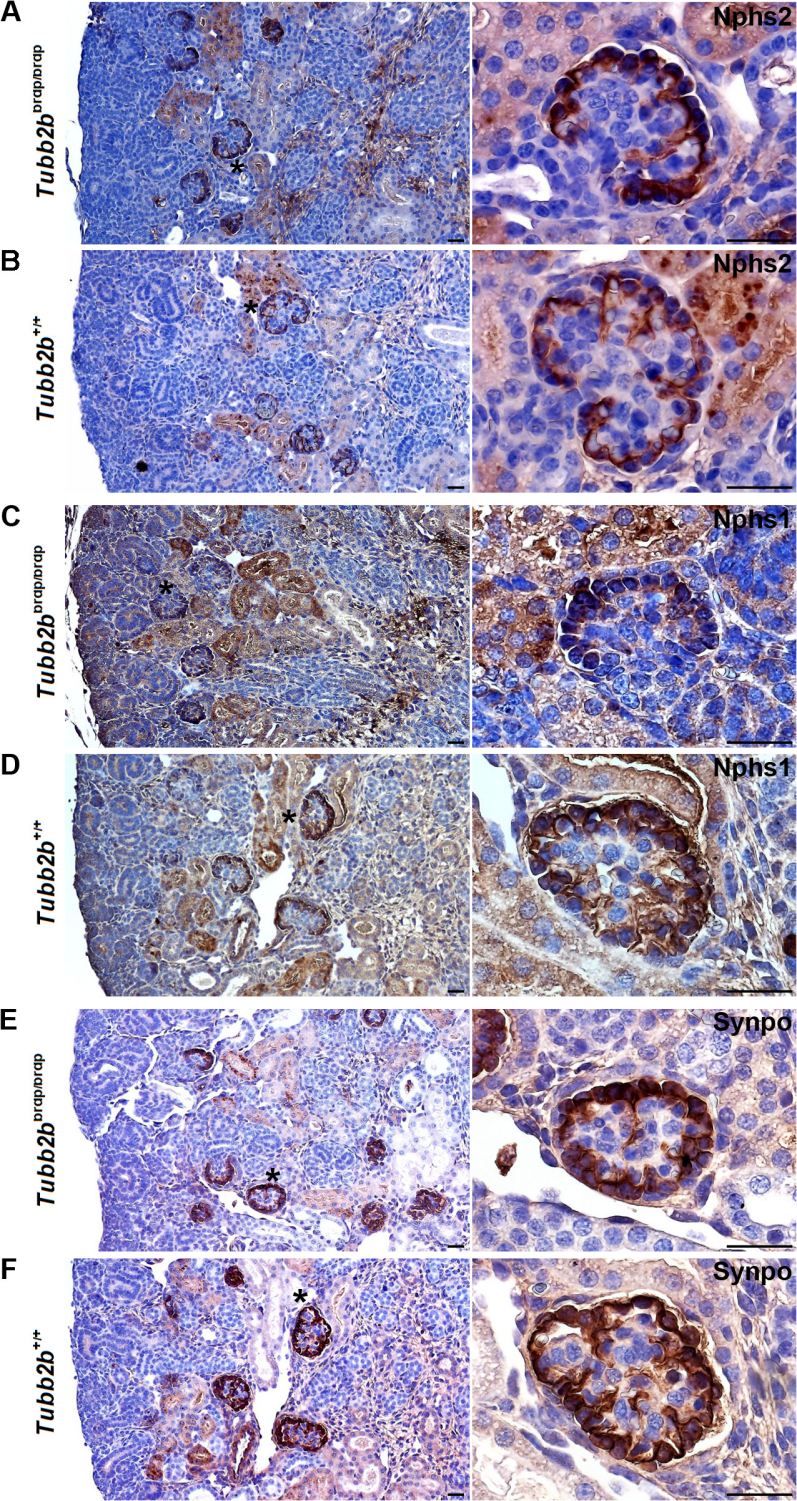
Nphs2-, Nphs1- and Synpo staining of wild type and *Tubb2b* ^brdp/brdp^ mouse kidneys (E18.5). Immunohistochemical staining of mice kidneys (dark-brown Nphs2, Nphs1 and Synpo stainings result from 3,3'-diaminobenzidine. Nuclei were stained with hematoxylin (blue). (A+B) Nphs2 staining. (A) *Tubb2b* ^brdp/brdp^ kidneys show a specific Nphs2 staining in podocytes arranged like a row of pearls at the periphery of the glomerulus. (B) Within the developing wild type kidney early capillary loop stages and maturing glomeruli are labeled. (C+D) Nphs1 staining. (E+F) Synpo staining. (C-F) Both, Nphs1 and Synpo patterns are comparable to the Nphs2 staining. Black asterisks: Enlarged section areas on the right. Scale bars = 50μm.

To underscore these observed defects in podocyte development we analyzed the expression of the podocyte-specific proteins Podocin (Nphs2), Nephrin (Nphs1) and Synaptopodin (Synpo) by immunohistochemistry (Fig 5). Nphs1 and Nphs2 expression in *Tubb2b* ^brdp/brdp^ kidneys was found to be elevated and localized to podocytes arranged like a row of pearls at the periphery of the glomerulus, further supporting a delay in podocyte maturation (Fig 5A+5C). In the developing wild type kidney Nphs2 and Nphs1 were detected in podocytes from the early capillary loop stage in the developing nephrons, and in mature glomeruli (Fig 5B+5D).

The Synpo expression pattern in the *Tubb2b* ^brdp/brdp^ kidneys was found to be similar to the Wt1, Nphs2 and Nphs1 staining, confirming the developmental defects seen in the mutant mice. (Fig 5E). As shown in Fig 5F, Synpo was observed in the wild type kidneys in the early capillary loop stage glomeruli and in glomeruli of later developmental stages.

**Fig 6:**
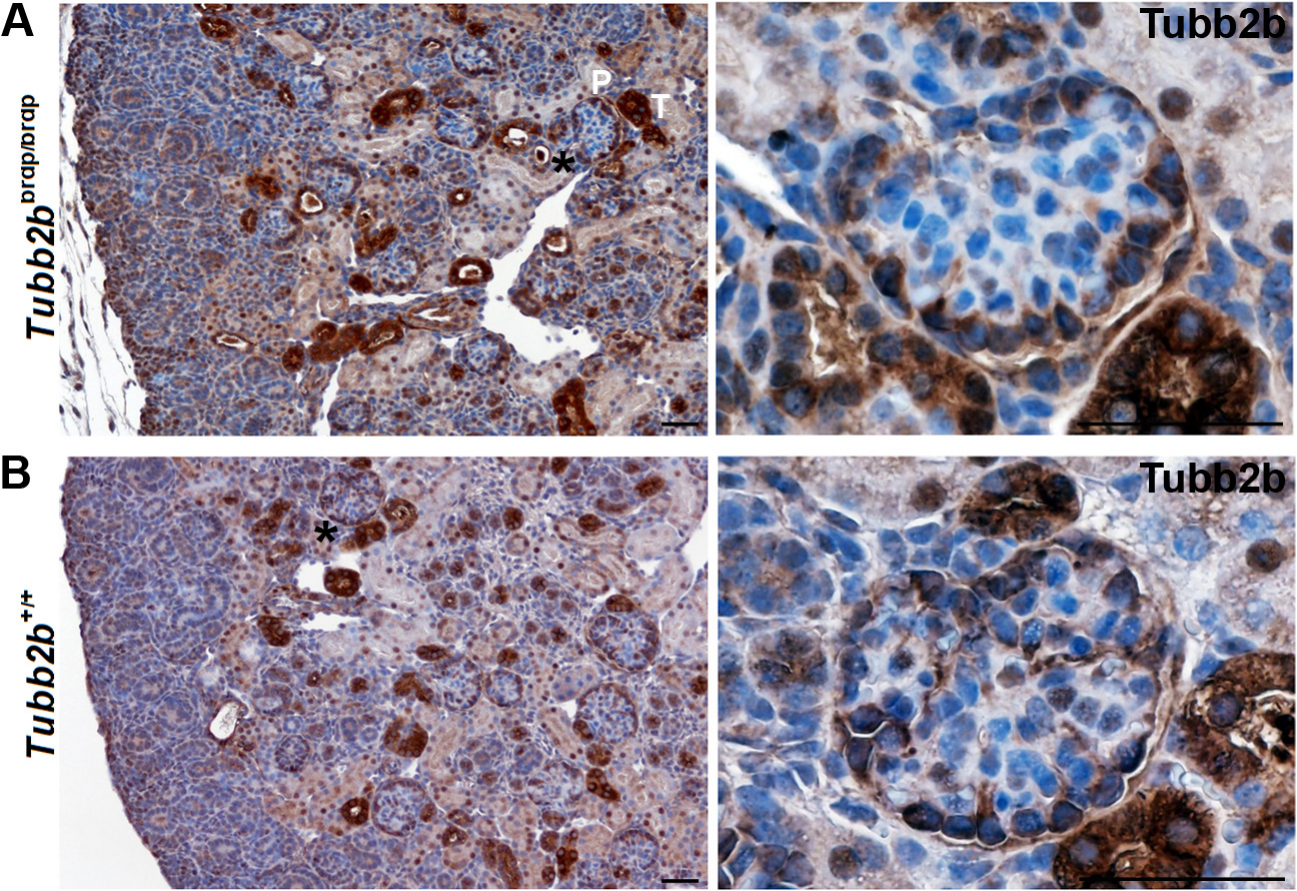
Tubb2b staining of wild type and *Tubb2b* ^brdp/brdp^ mouse kidneys (E18.5). Immunohistochemical staining of mice kidneys (dark-brown Tubb2b staining results from 3,3'-diaminobenzidine. Nuclei were stained with hematoxylin (blue). (A) *Tubb2b* ^brdp/brdp^ kidneys show a specific cytoplasmic Tubb2b expression in tubuli (T) and in podocytes (P), confirming the developmental defects seen with the Wt1, Nphs2, Nphs1 and Synpo stainings. (B) Tubb2b expression in wild type kidneys is restricted to the mature podocytes. Interestingly in the developing wild type kidney Tubb2b is not expressed in the early developmental stages of maturing podocytes. Note, that in murine podocytes nuclear Tubb2b seems much less expressed compared to human kidneys. Black asterisks: Enlarged section areas on the right. Scale bars = 50 μm.

Similar to Wt1, Nphs2, Nphs1 and Synpo, expression of Tubb2b can be found in podocytes organized like pearls at the periphery of the glomerulus, further establishing the developmental defects in *Tubb2b* ^brdp/brdp^ mice (Fig 6A). In wild type kidneys, Tubb2b is not expressed at early stages of podocyte development but restricted to mature podocytes (Fig 6B).

**Fig 7:**
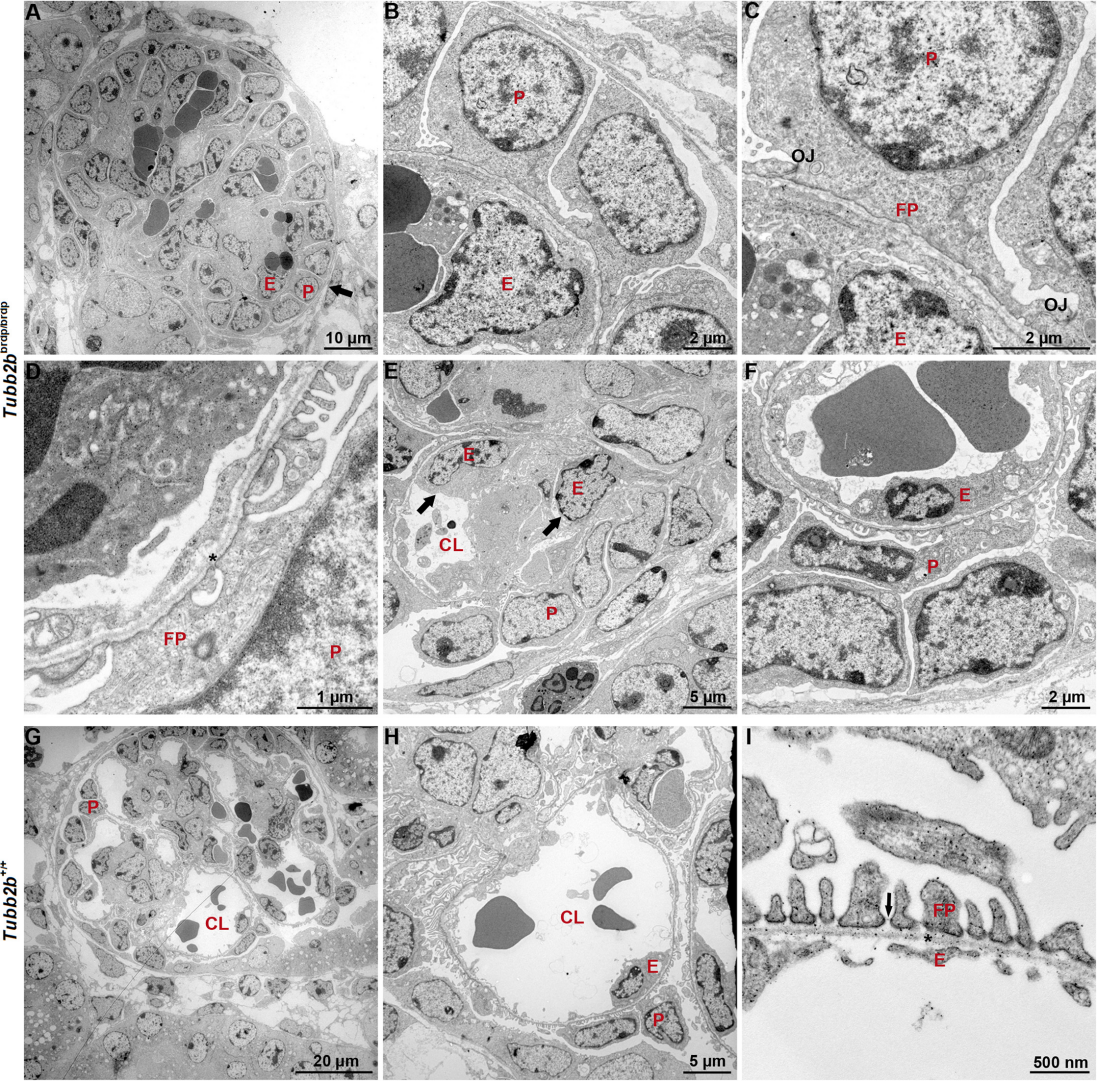
Transmission electron micrographs of wild type and *Tubb2b* ^brdp/brdp^ mouse kidneys (E18.5). (A-F) With TEM, a delay in glomerular endothelial (E) and podocyte (P) development could be observed in *Tubb2b* ^brdp/brdp^ mice. (A+B) Glomerulus with immature, cuboidal podocytes (black arrow). (C+D) Incompletely differentiated podocyte foot processes (FPs); some are extremely wide and linked by occludens junctions (OJs). (D) The glomerular basement membrane (asterisk) appears to be normal. (A+E) Glomeruli with no or small visible capillary lumen (CL) and multiple endothelial cells within the capillary loops (black arrows). (F) Swollen and vacuolated glomerular endothelial cells with decreased fenestrations. (G-I) Glomeruli of wild type *Tubb2b* mice had open glomerular capillaries with fenestrated endothelium, differentiated podocyte foot processes linked by SDs (black arrow) and a normal glomerular basement membrane.

On examination with TEM, significant morphologic changes of foot process and glomerular endothelium development were observed in *Tubb2b* ^brdp/brdp^ glomeruli (Fig 7). Some glomeruli from *Tubb2b* ^brdp/brdp^ mice had immature, cuboidal podocytes (Fig 7A+7B). In some areas, podocyte foot processes appeared incompletely differentiated. They were extremely wide and linked by occludens junctions (OJs) rather than SDs (Fig 7C+7D). Often, there was no visible capillary lumen in *Tubb2b* ^brdp/brdp^ glomeruli, even in most mature glomeruli, and multiple endothelial cells were visualized within the capillary loops (Fig 7A+7E). In addition, glomerular endothelial cells were swollen and vacuolated, with decreased fenestrations (Fig 7F). The glomerular basement membrane in *Tubb2b* ^brdp/brdp^ mice appeared morphologically similar to that seen in controls (Fig 7D). Glomeruli of wild type *Tubb2b* ^+/+^ mice had, according to age, open glomerular capillaries with fenestrated endothelium, differentiated podocyte foot processes linked by SDs and a normal glomerular basement membrane (Fig 7G–7I).

Collectively, the observed delay in podocyte differentiation following alteration of Tubb2b function underscores a role of the MT protein TUBB2B during kidney organogenesis. The immature podocyte phenotype seems not to be due to a prolonged proliferation of the Wt1-positive podocyte progenitors at the comma- and S-shaped body stage as no difference in the expression of the proliferation marker MKI67 could be observed (data not shown). These findings and the delay in capillary development remains to be studied more precisely in future experiments.

## Discussion

The immense variation in podocyte cell shape required for proper podocyte function depends on the underlying network of dynamic and interconnected actin and MT polymers. Glomerular diseases are often associated with foot process effacement and podocytopenia leading to severe proteinuria, and are closely linked to disaggregation and altered distribution of AFs [24]. Podocytes *in vivo* display typical arborized morphology with two types of processes–thick primary foot processes containing MTs as well as IFs [25] and thin secondary foot processes in which AFs represent the core cytoskeletal elements [26]. The molecular mechanisms of the reorganization of the podocyte actin cytoskeleton have been well studied over the last years whereas the function of the primary foot processes as well as the role of MTs and IFs is still poorly understood. Previous experiments, both *in vivo* and *in vitro*, showed that MTs are essential not only for formation but also for maintenance of intact primary podocyte foot processes [17,18,27].

In the present study, we identified by differential gene expression analysis of EV treated podocytes in an experimental model of podocyte injury a large number of genes being involved in cytoskeletal organization and stability comprising MT and actin-associated genes. In particular we observed a significant increase in the expression of *TUBB*, *TUBB2B* and *TUBA1A*, three major components of MTs. Interestingly a podocyte-specific expression of TUBB2B was observed in human and mouse podocytes.

MTs are produced by polymerized heterodimers of alpha- and beta-tubulin, encoded by tubulin genes which share substantial levels of sequence homology and are regulated spatially and temporally [28,29]. The function of MTs has been extensively studied in neurons. Here, they are crucial for neurite initiation, cortical development, cortical malformations and neuronal migration [30–32]. Functional studies suggest that abnormal neuronal migration would result from defective interactions in the tubulin heterodimer assembly and altered three-dimensional conformation of the tubulins, with compromised interaction with MT associated proteins or MT motor proteins [33]. Recently, mutations in neuronal tubulin genes (e.g. TUBA1A, TUBB2B, TUBB3 and TUBA8) have been identified in patients suffering from nervous system disorders, underlining the importance of tubulin isotypes in MT dynamics during brain development and axon guidance [34,35]. Overall, the identified *TUBB2B* mutations are suggested to alter the proper functions of MTs but the precise molecular function of TUBB2B in cortical development is still unclear. Stottmann and coworkers describe neurodevelopmental defects in mice with a homozygous missense mutation in *Tubb2b* due to a major increase in apoptosis and abnormal proliferation of the basal progenitors [19]. Besides these well-studied effects in the brain, podocyte-specific functions of TUBB2B have not been analyzed yet.

In podocytes, MTs are the scaffold of primary foot processes and the central cell body [25,36–40]. Disruption of MT elongation with vinblastine resulted in severe damage of primary foot processes *in vivo* [17,37,41] and blocked foot process formation *in vitro* [18].

In the present study, analyses of kidneys from *Tubb2b* ^brdp/brdp^ mice by the use of the podocyte-specific markers Wt1, Nphs2, Nphs1 and Synpo together with the Tubb2b expression pattern showed an abnormal podocyte morphology consistent with a delay in kidney development. A MT associated migratory defect of premature glomerular and perhaps endothelial cells might underlie this phenomenon, which is a focus of ongoing experimental studies.

Besides TUBB2B the MT associated protein DCDC2 was identified as a novel gene in podocytes, differentially regulated by mTOR inhibition. DCDC2 belongs to the superfamily of doublecortin (DCX) domain containing proteins. It regulates cytoskeletal dynamics by binding to and stabilizing MTs by linking adjacent tubulin protofilaments and has been identified in primary cilia of neuronal cells [42–50]. Kobayashi and colleagues demonstrated that MT associated proteins cause MT-based foot process formation *in vitro* via enhancement of MT assembly as well as an increase in MT stability [51]. DCX-microtubule effects have been extensively studied in neurons, including recent studies revealing cooperative binding effects, changes in MT structure, and regulation of molecular motors [48,49,52–54]. Mutations in the human *DCX* gene, the first characterized gene of the family, result in abnormal neuronal migration, epilepsy, mental retardation and cause double cortex syndrome and lissencephaly in humans [43]. Downregulation of *DCX* by RNA interference in animal models leads to neuronal migration disorders similar to those seen in the brains of dyslexic individuals [55,56]. Very recently, a novel clinical entity has been linked to recessive human *DCDC2* mutations with disruption of Wnt signaling causing a form of renal hepatic nephronophthisis-related ciliopathy [57]. In this work of Schueler et al., DCDC2 protein has been identified to colocalize with alpha-tubulin to the axoneme of primary cilia of human renal tubule cells and cholangiocytes in human liver and to multiciliated ependymal cells and pia mater cells in mouse brain. This is the first description of DCDC2 expression in the kidney, however the cellular function of DCDC2 in podocytes remains unclear. Interestingly, a homozygous human mutation in another ciliary gene *TTC21B* has lately been implicated in a familial form of focal-segmental glomerulosclerosis, clinically characterized by nephrotic-range proteinuria and severe podocyte damage [58]. In this work, the *TTC21B* gene product IFT139 was redistributed along the microtubule network in mature podocytes. These findings link defective functions of MTs and associated ciliary proteins to clinically relevant proteinuric disease.

Overall, this data is in line with the results of the present study demonstrating an up-regulation of TUBB2B and the MT associated protein DCDC2 as a consequence of mTORi-related rescue in damaged human podocytes. We here report for the first time an expression of TUBB2B and DCDC2 in human and mouse podocytes highlighting their potential relevance for the MT network and the actin cytoskeleton. Further studies will evaluate the role of TUBB2B and DCDC2 in human proteinuric disease.

## Supporting Information

S1 Fig Negative immunohistochemistry control. Negative control for TUBB2B and DCDC2 antibodies is performed without the primary antibody. 3,3'-diaminobenzidine (DAB) was used as chromogen (brown staining) and nuclei were stained with hematoxylin (blue). Scale bar = 50 μm.
(TIF)

S1 Table Affymetrix gene expression data. Fold change (FC) > 2.0 and p-value (p) < 0.05 for both comparisons. Not included data: Unknown gene annotations, chromosome open reading frames, family with sequence similarities, hypothetical proteins, genes with the same fold change tendency for both comparisons. PAN = puromycin aminonucleoside. EV = everolimus. MeOH = methanol, solvent for EV.
(DOCX)
