# Supplementary figures and images for: Everolimus Stabilizes Podocyte Microtubules via Enhancing TUBB2B and DCDC2 Expression
Source: PLoS One. 2015 Sep 2;10(9):e0137043. doi: 10.1371/journal.pone.0137043 (PMC4557973; doi:10.1371/journal.pone.0137043)

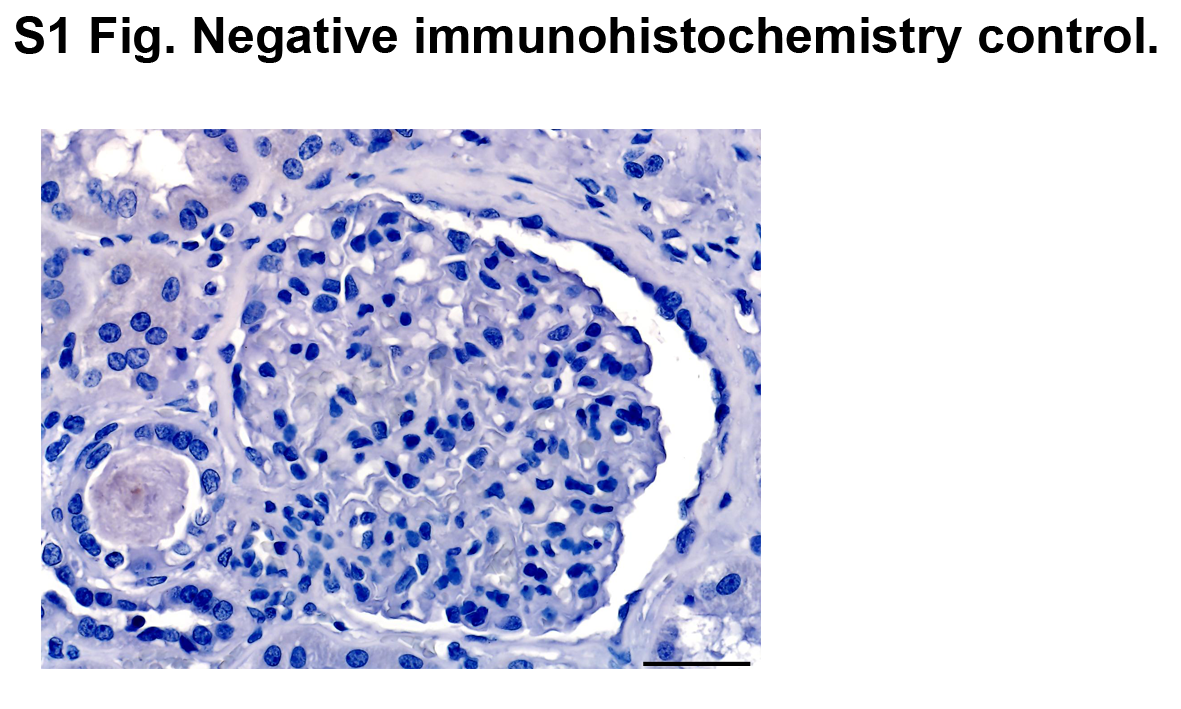

Supplement: S1 Fig — Negative control for TUBB2B and DCDC2 antibodies is performed without the primary antibody. 3,3'-diaminobenzidine (DAB) was used as chromogen (brown staining) and nuclei were stained with hematoxylin (blue). Scale bar = 50 μm. (TIF) [file pone.0137043.s001.tif]
