# Supplementary material for: Everolimus Stabilizes Podocyte Microtubules via Enhancing TUBB2B and DCDC2 Expression
Source: PLoS One. 2015 Sep 2;10(9):e0137043. doi: 10.1371/journal.pone.0137043 (PMC4557973; doi:10.1371/journal.pone.0137043)
Supplement: S1 Table — Fold change (FC) > 2.0 and p-value (p) < 0.05 for both comparisons. Not included data: Unknown gene annotations, chromosome open reading frames, family with sequence similarities, hypothetical proteins, genes with the same fold change tendency for both comparisons. PAN = puromycin aminonucleoside. EV = everolimus. MeOH = methanol, solvent for EV. (DOCX) [file pone.0137043.s002.docx]

**S1 Table. Affymetrix gene expression data.**
